# Naringin ameliorates the high glucose-induced rat mesangial cell inflammatory reaction by modulating the NLRP3 Inflammasome

**DOI:** 10.1186/s12906-018-2257-y

**Published:** 2018-06-22

**Authors:** Fenqin Chen, Guozhu Wei, Jiao Xu, Xiaoyu Ma, Qiuyue Wang

**Affiliations:** 1 Departments of Geriatric, the First Affiliated Hospital, China Medical University, Shenyang, 110001 China; 2 Department of Radiology, Orthopedic Hospital of Shenyang, Shenyang, 110001 China; 3 Departments of Endocrinology, the First Affiliated Hospital, China Medical University, Shenyang, 110001 China

**Keywords:** Diabetes mellitus, Diabetic kidney disease, NLRP3 inflammasome, NLRP3-caspase-1-IL-1β/IL-18 signaling pathway, Naringin

## Abstract

**Background:**

The Nucleotide binding and oligomerization domain-like receptorfamily pyrin domain-containing 3 (NLRP3)-inflammasome plays an important role in various diseases, including a variety of kidney diseases. Naringin exhibits anti-inflammatory and anti-oxidation effects among others, but its specific mechanisms are not clear. We investigated the expression of the NLRP3-inflammasome under high-glucose conditions, assessed the effects of naringin on that process, and further elucidated the role of naringin in the pathogenesis of diabetic kidney disease(DKD).

**Methods:**

To assess the therapeutic potential of naringin and the mechanisms involved, we cultured rat glomerular mesangial cells and grouped them according to different glucose concentrations, different action times, different concentrations of MCC950, and different concentrations of naringin.The cell proliferation was measured by MTT assay. The expression of Interleukin-1β(IL-1β) and Interleukin18 (IL-18) in the cell supernatant were detected by ELISA. The expression and activity of NLPR3, apoptosis-associated speck-like protein containing a caspase recruitment domain (ASC) and Caspase-1 were detected by Western Blot.

**Results:**

The expressions of NLRP3, ASC, caspase-1, IL-1β, and IL-18 in rat glomerular mesangial cells were significantly higher in the high glucose (HG) group than in the control normal glucose (NG) group and exhibited time-dependence activity. The expression levels of NLRP3, caspase-1, IL-1β, and IL-18 in different treatment groups were significantly lower compared with the HG group after 48 h of MCC950 pre-treatment (*p* < 0.05). Pre-treatment with naringin produced the same results. Naringin also inhibited the proliferation of cells.

**Conclusions:**

The NLRP3-inflammasome potentially plays a role in the process of activation and inflammation of glomerular mesangial cells as induced by high-glucose conditions. Naringin inhibited the proliferation of cells that were induced by high glucose. Further, it reduced the expression of inflammatory factors that are mediated by NLRP3 through the NLRP3-caspase-1-IL-1β/IL-18 signaling pathway, which makes naringin a potentially novel treatment for DKD disease.

**Electronic supplementary material:**

The online version of this article (10.1186/s12906-018-2257-y) contains supplementary material, which is available to authorized users.

## Background

Diabetic kidney disease (DKD) is one of the most serious chronic complications of diabetes mellitus (DM), is a strong risk factor for cardiovascular disease, and is a major cause of end stage kidney disease [1, 2]. The pathogenesis of DKD is complex, and there are no effective measures to treat it currently [3–6]. Although many therapeutic remedies focusing on hyperglycemia and high blood pressure have been used, many patients still suffer progressive and severe renal injury. Thus, the investigation of other pathogenic pathways and relevant therapeutic strategies is worthwhile.

Many studies have shown that chronic inflammation plays an important role in the progression of DKD [7–13], but the underlying mechanisms and specific therapies to target inflammation are lacking. There are two different mammalian immune systems, the innate system and the adaptive system. The innate immune system is the first line of protection against invading microorganisms, and the pyrin domain-containing-3 (NLRP3) inflammasome is an important component of it. The NLRP3 inflammasome is a member of the nucleotide-binding domain and leucine-rich repeat containing family, and is composed of NLRP3, apoptosis-associated specklike protein containing a CARD (ASC), and procaspase-1. It senses endogenous and exogenous danger signals, including lipopolysaccharides (LPS) and high glucose levels (HG), and then it cleaves procaspase-1 and activates the cytokines IL-1β, IL-18, and IL-33 that ultimately trigger the inflammatory cascade [14–16].

For patients with chronic kidney disease, the NLRP3 inflammasome may be a potential therapeutic target [17, 18]. A decline in renal function is related to the accumulation of inflammatory cells in the kidneys, which suggests a causative link. In addition, inhibition of inflammatory cell recruitment into the kidneys has been shown to be protective in experimental DKD [19, 20].

Naringin (4,5,7-trihy droxyflavanon e-7-rhamnoglucoside) is a bioflavonoid. It is derived from grapefruit and other related citrus fruit species and has been reported to possess antioxidant, free radical scavenging, and metal-chelating properties. Naringin has also been reported to have therapeutic activities such as anti-inflammatory, anti-apoptotic, anticancer, and cardioprotective effects [21]. It has also been reported to lower glucose levels [22–24]. However, in treating diabetic complications, the role of naringin has not been investigated. The aim of this study was to investigate the expression of the NLRP3 inflammasome under high glucose conditions, the effects of naringin under these conditions, and the effects of naringin on the pathogenesis of DKD (Additional file 1).

## Methods

### Reagents

Rat mesangial cells (HBZY-1) were obtained from the Wuhan cell repository (Wuhan, China). Naringin (purity of ≥98%) was obtained from Cayman (CA, USA), stored at 2–4 °C, and protected from light. MCC950 was purchased from Selleckchem (CA, USA). IL-1β and IL-18 ELISA kits were purchased from R&D Systems (USA). Rabbit anti-NLRP3 and rabbit anti-ASC were obtained from Cell Signaling Technology (CST; CA, USA). Rabbit anti-caspase-1 and GAPDH were obtained from Santa Cruz (CA, USA).

### Cell culture and experimental design

Rat glomerular mesangial cells were cultured in Dulbecco’s Modified Eagle Medium (DMEM) containing 5.6 mmol/L glucose and 10% fetal bovine serum (FBS) at 37 °C with 5% CO_2_ and 95% air. Rat glomerular mesangial cells were routinely cultured and grouped according to different experimental objectives: (1) According to different glucose concentrations (all at 48 h): normal control group (NG, glucose concentration of 5.6 mmol) and high glucose groups (glucose concentrations of 15, 25, 30, 35, and 50 mmol/L); (2) According to different action times: NG, high glucose group (HG, glucose concentration of 30 mmol/L), and mannitol osmotic pressure control group (HM, glucose concentration of 5.6 mmol/L and mannitol concentration of 24.4 mmol/L), with action times of 24, 48, and 72 h; (3) According to different concentrations of MCC950 (all at 48 h): NG, HG, and MCC950 therapy groups (the concentrations of MCC950 were 1,10,50 or 100 μmol/L); (4) According to naringin concentration (all at 48 h): NG, HG, and naringin therapy groups (initial concentrations of naringin were 0.01,0.1,1,10,100 μmol/L, and the second concentrations of naringin were 5,10, 20,40 or 80 μmol/L); (5) According to different intervention factors (all at 48 h): NG, HG, HG + naringin, HG + MCC950.

### Detection of cell proliferation (MTT method)

Rat glomerular mesangial cells were seeded in 96-well plates at a density of 5–10 × 10^3^ cells/well and cultured in complete DMEM. When cells were 70 to 80% confluent, they were incubated overnight with 1640 medium without serum. Three to five wells were included for each predefined group. Additions were then added for experimental treatments with different amounts of high glucose medium or different concentrations of naringin solution, and cultures were continued for 24, 48, and 72 h. Ten microliters of MTT solution (solubility of 5 mg/ml) was added to each well and then incubated for 4 h. After the MTT solution was discarded, DMSO was added to each well and incubated at room temperature for 10 min. Finally, absorbance was measured with a microplate reader at a wavelength of 550 nm.

### Western blotting

Total protein was extracted from glomerular mesangial cells using a protein extraction kit (Biyuntian, Beijing, China). Proteins were then separated by sodium dodecylsulfate-polyacrylamide gel electrophoresis (SDS-PAGE) and transferred to a polyvinylidene difluoride (PVDF) membrane (Millipore). Immunoblotting was performed using anti-NLRP3 antibody (rabbit; 1:1000; CST), anti-ASC antibody (rabbit; 1: 1000; CST), anti-caspase-1 antibody (rabbit; 1:1000; Santa Cruz), and anti-GAPDH antibody (rabbit; 1:1000; Santa Cruz).

### ELISA of IL-1β and IL-18 protein synthesis in cell culture supernatants

Cells culture supernatants were collected and stored at − 70 °C until further testing. Protein levels of IL-1β and IL-18 in culture supernatants were determined using a commercial ELISA assay kit (R&D Systems) according to the manufacturer’s instructions.

### Statistics

Data are presented as means ± SEM. Statistical significance was determined using One Way Analysis of Variance (ANOVA) tests followed by post hoc Bonferroni’s correction using Graph Pad Prism 5.0 software. A *p*-value of less than 5% (*p* < 0.05) was considered statistically significant.

## Results

### Effect of glucose concentrations on the proliferation of rat glomerular mesangial cells at different time points

**Fig. 1:**
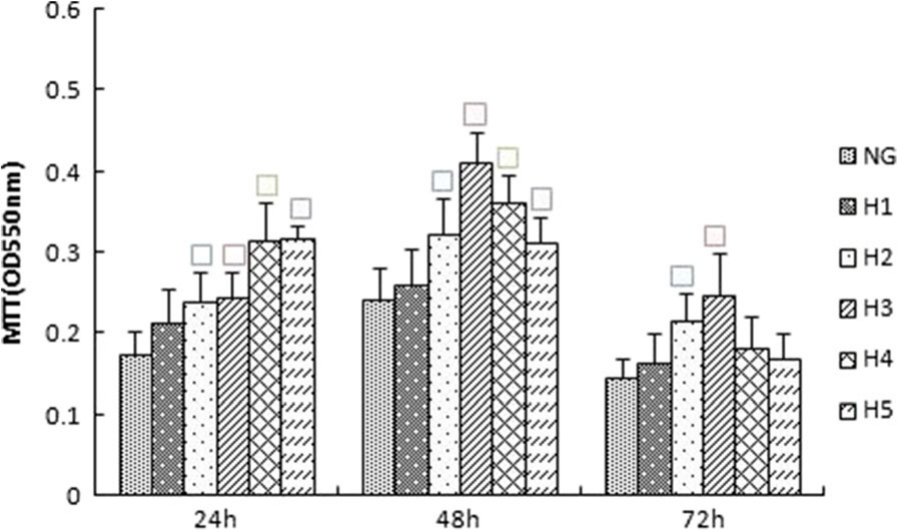
Effects of different concentrations of glucose on the proliferation of rat glomerular mesangial cells at various time points (24 h,48 h,72 h).Proliferation was measured by MTT. NG:normal control group,H1:glucose concentration of 15 mmol/L,H2:glucose concentration of 25 mmol/L,H3:glucose concentration of 30 mmol/L,H4:glucose concentration of 35 mmol/L,H5:glucose concentration of 50 mmol/L.Data are presented in mean ± SD,^□^*P* < 0. 05 versus normal control group

To investigate the effect of high glucose concentrations on rat glomerular mesangial cells over time, we performed experiments with varying concentrations of glucose and sampled at varying time points. We found that cellular absorbance values increased in a dose-dependent manner (*p* < 0.05) over multiple glucose concentrations (5.5, 15, 25, 30, 35 and 50 mmol/L) sampled at 24, 48, and 72 h. The absorbance of cells within the 15 mmol/L group was higher than that of the NG group, but there was no significant difference between the groups (*p* > 0.05). Compared to the NG group, the high-glucose group exhibited increased proliferation, with rates of OD value increases significantly peaking at 48 h. Cell proliferation slowed at 72 h (Fig. 1). These results suggest that when glucose increases over a certain time, cell proliferation increases but ultimately levels off.

### Effects of different concentrations of glucose on the expression of NLRP3

**Fig. 2:**
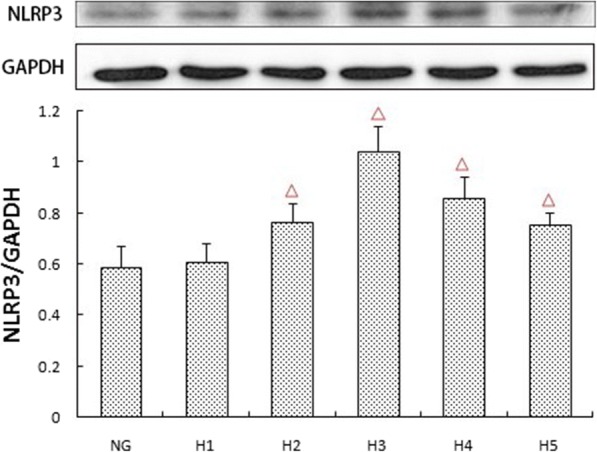
Effects of different concentrations of glucose on the protein expression of NLRP3.The protein was measured by western blotting.GAPDH was used as an internal loading control. NG:normal control group,H1:glucose concentration of 15 mmol/L,H2:glucose concentration of 25 mmol/L,H3:glucose concentration of 30 mmol/L,H4:glucose concentration of 35 mmol/L,H5:glucose concentration of 50 mmol/L.Data are presented in mean ± SD,^△^*P* < 0. 05 versus normal control group

The expression of NLRP3 in cultured rat glomerular mesangial cells increased with different glucose concentrations after 48 h, with the highest values in the 30 mM glucose stimulation treatment, which was significantly higher than the control group (*p* < 0.05) (Fig. 2). These results formed the basis for subsequent experimentation of glucose concentrations.

### Expression of NLRP3, ASC, caspase-1, IL-1β, and IL-18 in each group at different time points

**Fig. 3:**
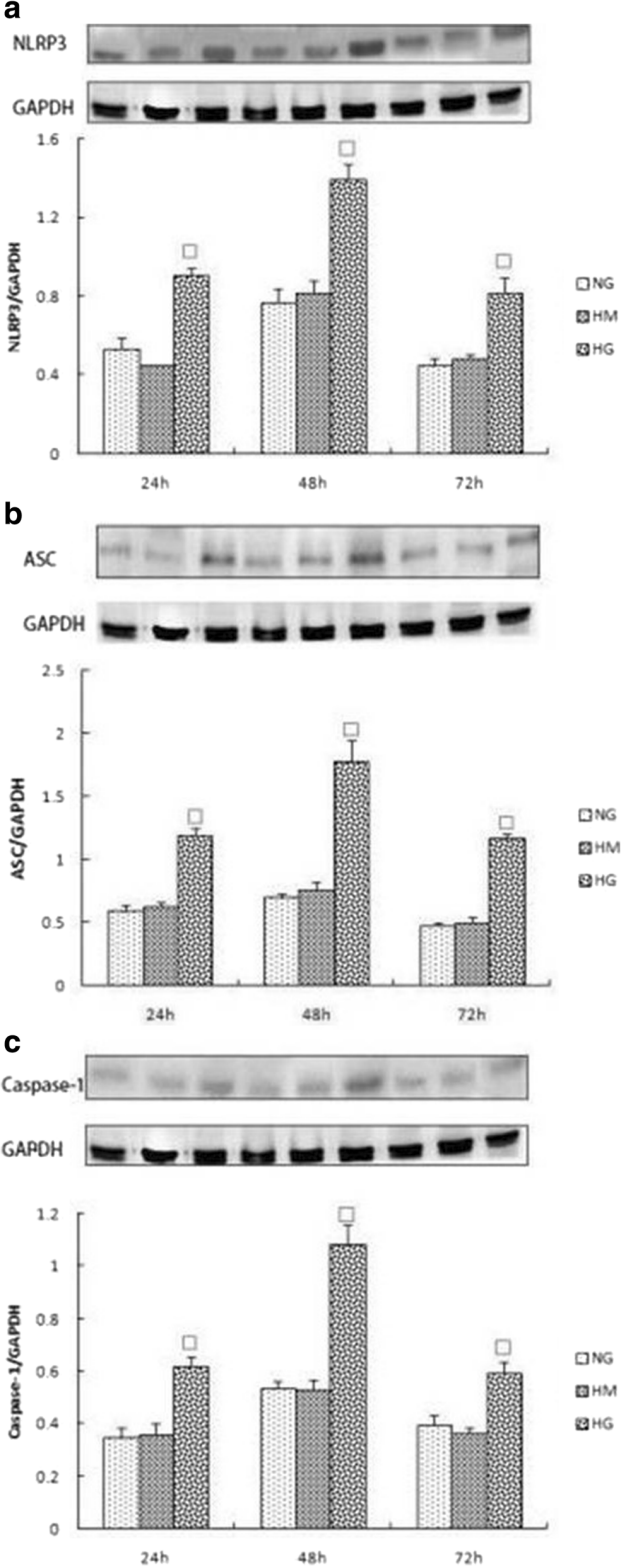
**a**, **b**, **c** Expression of NLRP3, ASC and Caspase-1 in NG,HM,HG group at various time points (24 h,48 h,72 h).The protein was measured by western blotting.GAPDH was used as an internal loading control. NG:normal control group,HM:mannitol osmotic pressure control group,HG:high glucose group.Data are presented in mean ± SD, ^□^*P* < 0. 05 versus normal control group

**Fig. 4:**
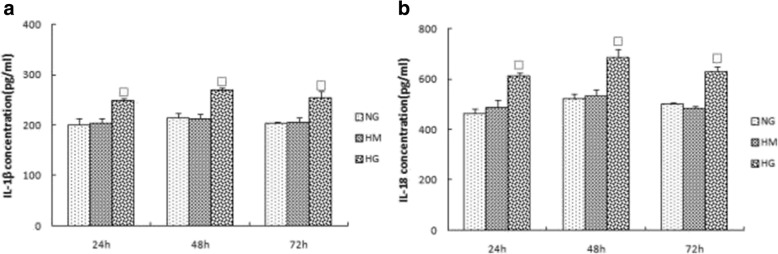
**a**, **b** Expression of IL-1β and IL-18 in NG,HM,HG group at various time points (24 h,48 h,72 h).The protein levels of IL-1β and IL-18 in culture supernatants were determined by ELISA. NG:normal control group,HM:mannitol osmotic pressure control group,HG:high glucose group.Data are presented in mean ± SD, ^□^*P* < 0. 05 versus normal control group

We further explored whether high glucose can induce activation of inflammasomes in rat glomerular mesangial cells by analyzing the expression of NLRP3, ASC, and caspase-1. Within 48 h, inflammasome response was activated in a time-dependent manner. Inflammasome activity decreased when culturing to 72 h (Fig. 3a, b, c). IL-1β and IL-18 in the supernatant increased at 24 h, and their expression peaked at 48 h (*p* < 0.05) (Fig. 4a, b). To assess whether osmotic pressure alone had an effect on the inflammasome and cytokines, we used mannitol to stimulate rat glomerular mesangial cells and found no significant effect.

### Effects of MCC950 on the expression of NLRP3, caspase-1, IL-1β, and IL-18

**Fig. 5:**
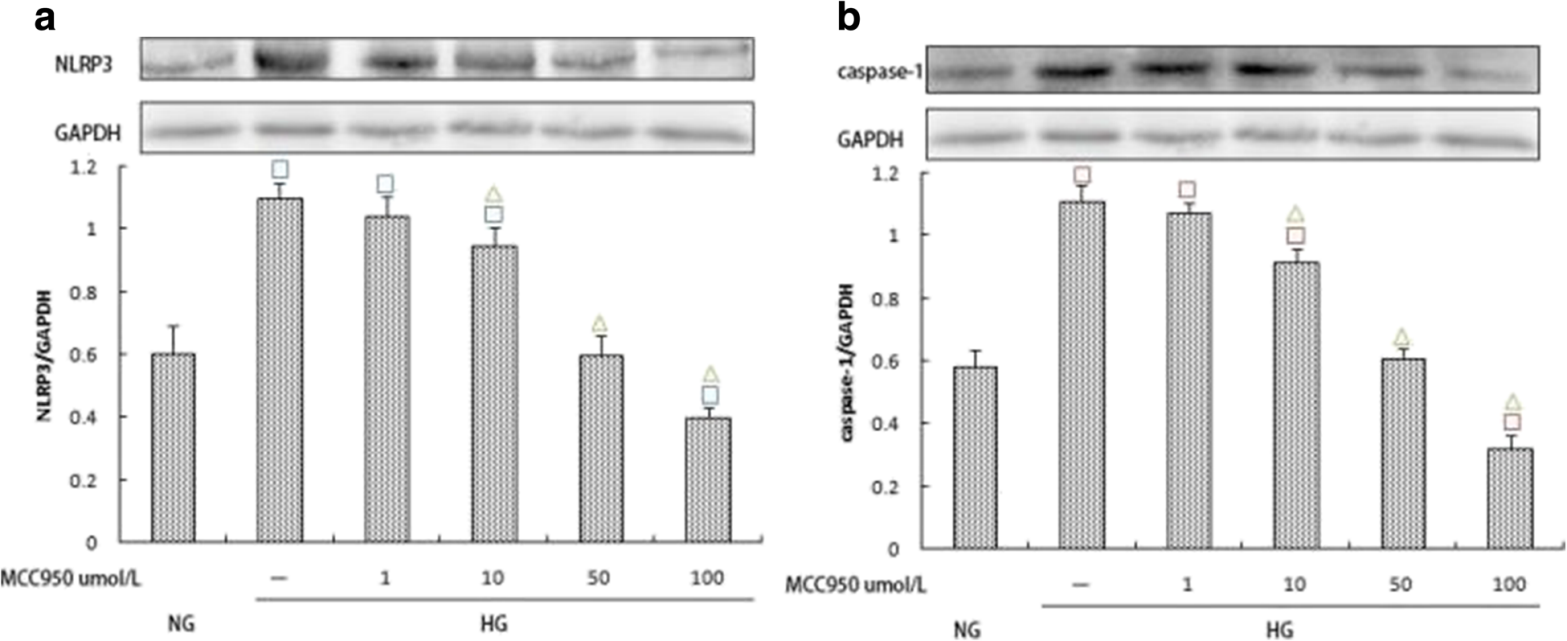
**a**, **b** Effects of varying MCC950 concentrations (0,1,10,50 and 100 μmol/L) on NLRP3 and Caspase-1 expression within the high glucose group. The protein was measured by western blotting.GAPDH was used as an internal loading control. NG:normal control group,HG:high glucose group.Data are presented in mean ± SD, ^□^*P* < 0. 05 versus normal control group, ^△^*P* < 0. 05 versus high glucose group

**Fig. 6:**
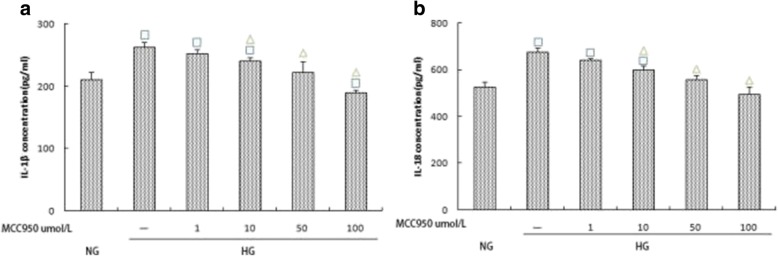
**a**, **b** Effects of varying MCC950 concentrations (0,1,10,50 and 100 μmol/L) on IL-1β and IL-18 expression within the high glucose group. The protein levels of IL-1β and IL-18 in culture supernatants were determined by ELISA. NG:normal control group,HG:high glucose group.Data are presented in mean ± SD, ^□^*P* < 0. 05 versus normal control group, ^△^*P* < 0. 05 versus high glucose group

To investigate the effect of high glucose concentrations on the expression of IL-1β and IL-18 by activating caspase-1 through NLRP3 in rat glomerular mesangial cells, we used the NLRP3-specific inhibitor MCC950 to treat cells within the high glucose group. Western blotting was used to assess the expression of NLRP3 and caspase-1 under different concentrations of MCC950. MCC950 at varying concentrations inhibited the expression of NLRP3 and caspase-1 in addition to the expression of IL-1β and IL-18. Differences in groups with varying MCC950 concentrations (10, 50, and 100 μmol/L) were significant (*p* < 0.05) (Fig. 5a, b; Fig. 6a, b). These results suggest that MCC950 inhibits elevated glucose-induced activation of NLRP3, which then results in decreased expression of IL-1β and IL-18. Further, the results indicate that high glucose plays a role in inflammation partly by mediating the NLRP3-caspase-1-IL-1β/IL-18 signaling pathway.

### Effects of different naringin concentrations on the activity and proliferation of rat glomerular mesangial cells under normal and high glucose conditions

**Fig. 7:**
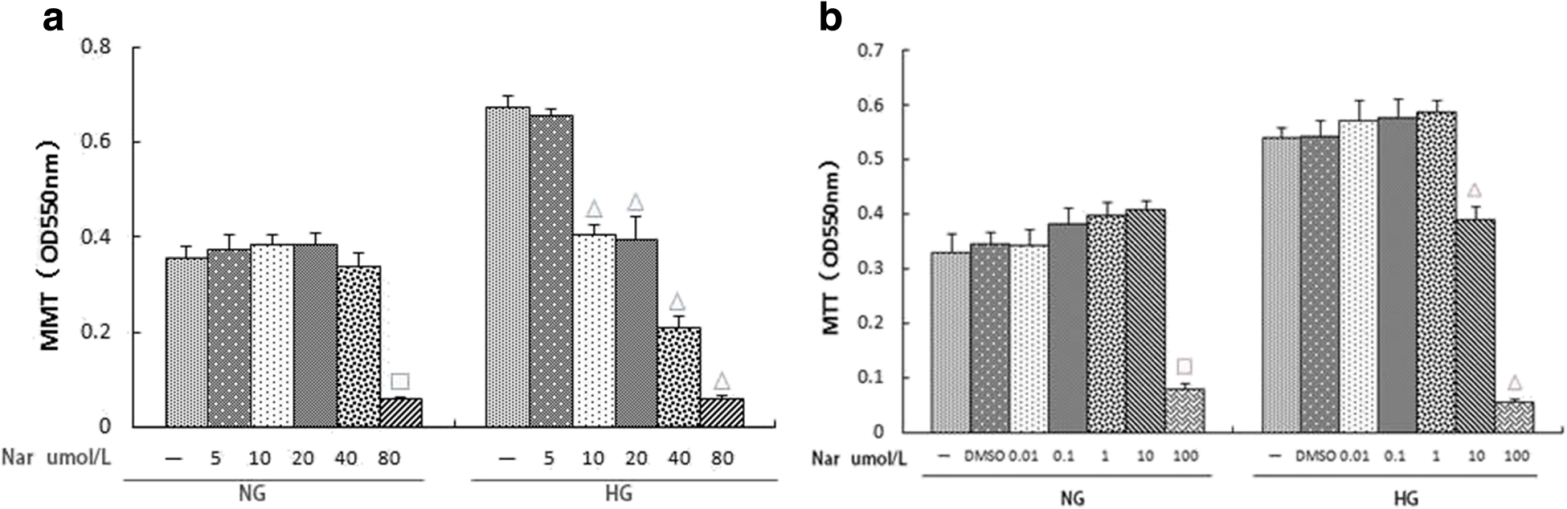
**a**, **b** Effects of (**a**) different naringin concentrations (0,0.01,0.1, 1.00,10.00 and 100umol/L),(**b**) different naringin concentrations (0,5,10,20,40 and 80 umol/L) on the activity of normal glucose and proliferation of high glucose in rat glomerular mesangial cell.Proliferation was measured by MTT. NG:normal control group,HG:high glucose group,DMSO:DMSO content of 1% group.Data are presented in mean ± SD, ^□^*P* < 0. 05 versus normal control group, ^△^*P* < 0. 05 versus high glucose group

To determine whether naringin is toxic to normal cultured rat glomerular mesangial cells, we exposed normally cultured rat glomerular mesangial cells to different concentrations of naringin and assayed them by MTT. Cell viability was not significantly different in the naringin groups compared to the NG control group, except for the 100 μmol/L naringin group. Cells with 1% DMSO added exhibited similar results to the NG group. These results indicated that when the concentration of naringin was 0.01,0.10,1.00 or 10.00 μmol/L, there was no toxic effect on cell viability (Fig. 7a).

Previous studies have indicated that high glucose concentrations can significantly promote the proliferation of mesangial cells. Based on this, we assessed the effect of naringin on the proliferation of rat glomerular mesangial cells under high glucose conditions. MTT assays indicated that the inhibitory effect on the proliferation of high glucose-induced cells gradually increased with increasing naringin concentration. Differences when the concentration of naringin was 10 μmol/L and 100 μmol/L were statistically significant (*p* < 0.05). There was no significant difference in the OD value between the 1% DMSO group and the HG group. These results showed that the inhibitory effect of naringin on high glucose-induced proliferation was dose-dependent in a dose range of 0.01–100 μmol/L (Fig. 7a). Moreover, the inhibitory effect of naringin at 100 μmol/L was not due to the toxicity of DMSO.

Based on these results, we refined the concentration of naringin and, using the same methods, found that a naringin concentration of 40 μmol/L had no effect on the activity of rat glomerular mesangial cells cultured under normal glucose conditions, while the dosage resulted in a significant inhibitory effect on the proliferation of high glucose-induced cells (Fig. 7b).

### Effects of different naringin concentrations on the expression of NLRP3, caspase-1, and IL-1β/IL-18

**Fig. 8:**
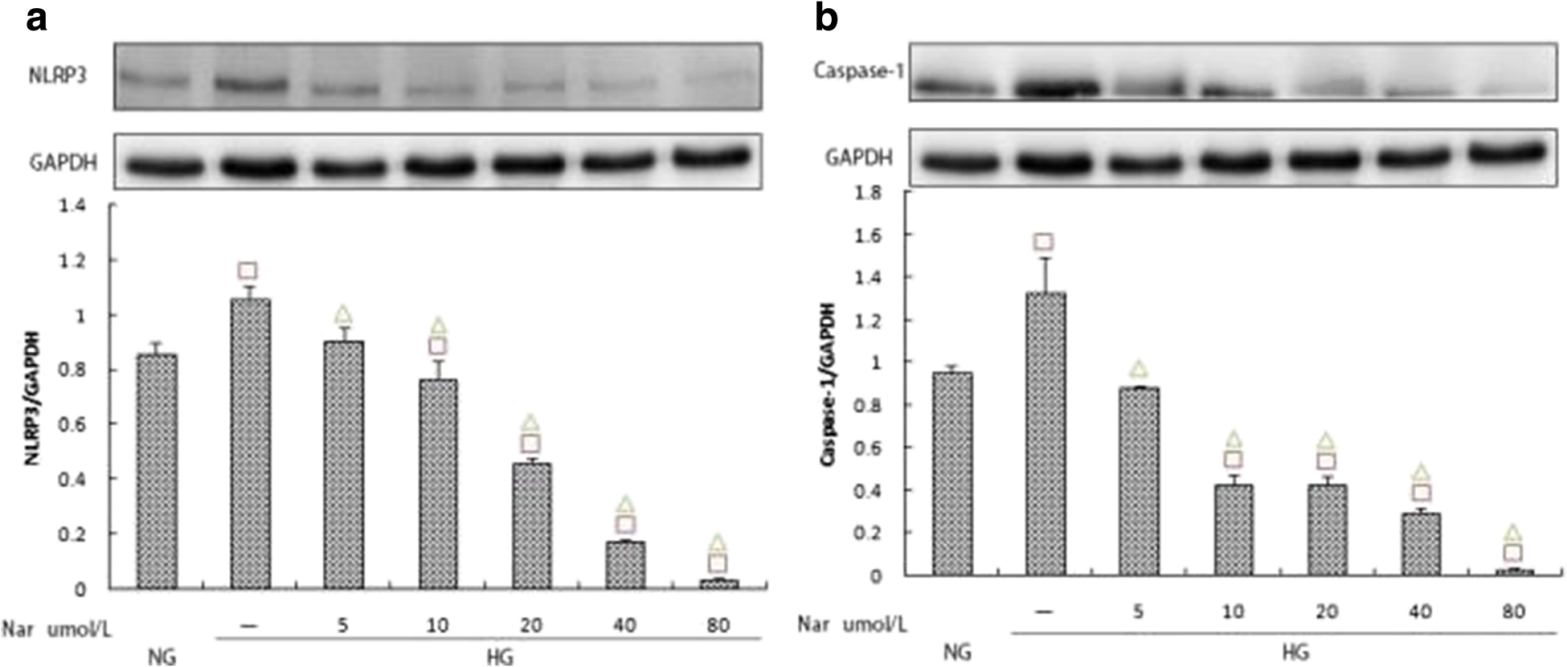
**a**, **b** Effects of different naringin concentrations (0,5,10,20,40 and 80 umol/L) on the expression of NLRP3 and Caspase-1 within the high glucose group. The protein was measured by western blotting.GAPDH was used as an internal loading control. NG:normal control group,HG:high glucose group.Data are presented in mean ± SD, ^□^*P* < 0. 05 versus normal control group, ^△^*P* < 0. 05 versus high glucose group

**Fig. 9:**
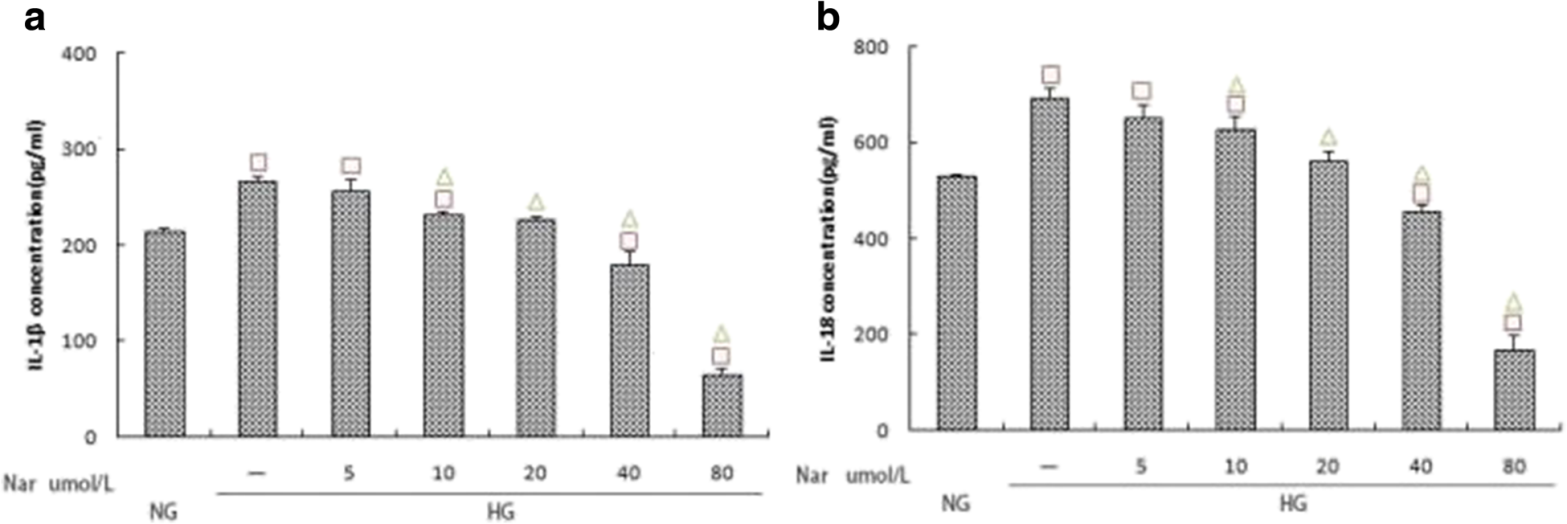
**a**, **b** Effects of different naringin concentrations (0,5,10,20,40 and 80 umol/L) on the expression of IL-1β and IL-18 within the high glucose group.The protein levels of IL-1β and IL-18 in culture supernatants were determined by ELISA. NG:normal control group,HG:high glucose group.Data are presented in mean ± SD, ^□^*P* < 0. 05 versus normal control group, ^△^*P* < 0. 05 versus high glucose group

To verify the MTT results, we used western blotting and ELISA to determine the inhibitory effect of naringin. Compared to the HG group, naringin treatment inhibited the expression of NLRP3, caspase-1, IL-1β, and IL-18, and the inhibitory effect was dose-dependent. Furthermore, the differences between naringin concentrations of 40 μmol/L and 80 μmol/L were statistically significant (*p* < 0.05) (Fig. 8a, b; Fig. 9a, b).

### Effects of different treatments on the expression of NLRP3, caspase-1, and IL-1β/IL-18

**Fig. 10:**
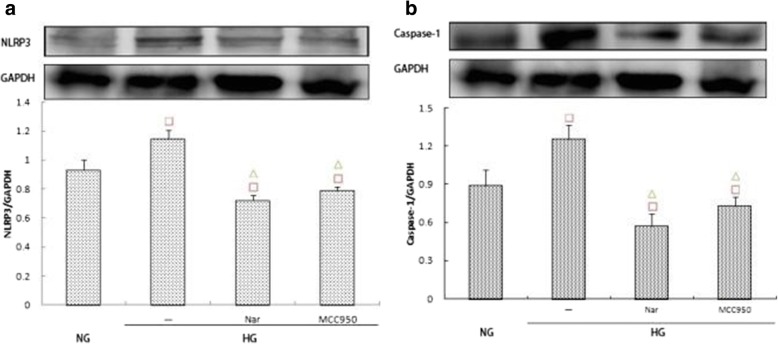
**a**, **b** Effects of naringin (40umol/L) and MCC-950 (100umol/L) on the expression of NLRP3 and Caspase-1 within the high glucose group. The protein was measured by western blotting.GAPDH was used as an internal loading control. NG:normal control group,HG:high glucose group,Nar:naringin 40umol/L + HG,MCC950:MCC-950 100umol/L + HG.Data are presented in mean ± SD, ^□^*P* < 0. 05 versus normal control group, ^△^*P* < 0. 05 versus high glucose group

**Fig. 11:**
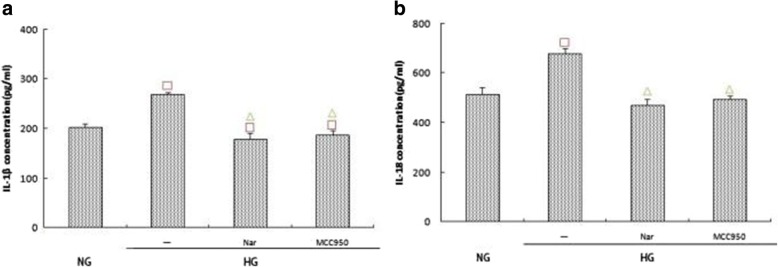
**a**, **b** Effects of naringin (40umol/L) and MCC-950 (100umol/L) on the expression of IL-1β and IL-18 within the high glucose group.The protein levels of IL-1β and IL-18 in culture supernatants were determined by ELISA. NG:normal control group,HG:high glucose group,Nar:naringin 40umol/L + HG,MCC950:MCC-950 100umol/L + HG.Data are presented in mean ± SD, ^□^*P* < 0. 05 versus normal control group, ^△^*P* < 0. 05 versus high glucose group

Western blot assays indicated that the expression of NLRP3 and caspase-1 in the HG + 40 μmol/L naringin group was significantly lower than that in the HG group (*p* < 0.05), which itself was significantly lower than that in the MCC950 group (Fig. 10a, b). ELISA further indicated that the secretion of IL-1β and IL-18 in the 40 μmol/L naringin group was significantly lower than that in the HG group, and the difference was statistically significant; additionally, there was less secretion than in the MCC950 group (Fig. 11a, b).

## Discussion

DKD is a chronic, low-grade inflammatory response that is characterized by the infiltration of immune cells and cytokines into kidney tissue. Immune-mediated inflammatory responses are a key component of DM and DKD. Inflammatory cytokines such as IL-1β and IL-18 are significantly elevated in renal tissue during DKD, and increasing the expression of these cytokines can improve kidney resistance to disease [19, 20]. Recent studies have shown that the NLRP3 inflammasome and IL-1β/IL-18 play important roles in gout, rheumatoid arthritis, atherosclerosis, DM, and oxalate-related nephropathy diseases [17, 25]. The roles of IL-1β and IL-18 in renal disease are well known, but the importance of inflammasomes in their activation has not been investigated. The relationship between the NLRP3 inflammasome and type 2 DM was first studied by the Tschopp laboratory who found that NLRP3 knockout mice were more tolerant of glucose concentration increases than wild type mice after 8 weeks of a high fat diet, and they were also more sensitive to insulin [26]. Following these results, further studies have reported on the relationship between NLRP3 and DKD [27–30]. These studies have indicated that NLRP3 and IL-1β/IL-18 increase to varying degrees in either DM animal models or high-glucose cell models, but their upstream or downstream relationships have not been investigated.

In this study, we found that the expression of NLRP3 increased with different increases of glucose concentration in rat glomerular mesangial cells, and it reached its highest expression when the glucose concentration was 30 mmol/L. These results indicated that high glucose levels can active the NLRP3 inflammasome. In addition, the expressions of NLRP3, ASC, caspase-1, IL-1β, and IL-18 were highest when glucose was at 30 mmol/L and within 48 h of experimental onset. Moreover, the expression levels of NLRP3, ASC, caspase-1, IL-1β, and IL-18 gradually increased in a time-dependent manner. Following these increases, and at 72 h, the expressions of NLRP3, ASC, caspase-1, IL-1β, and IL-18 gradually decreased, which was likely due to the consumption of glucose. The expressions of inflammatory cytokines in the mannitol osmolality group were not significantly different compared to the normal glucose control group, suggesting that the observed changes in expression were not related only to changes in osmotic pressure. It can thus be inferred that high glucose in DKD may mediate inflammatory responses by activating the NLRP3 inflammasome, which is consistent with the results of Feng et al. [19].

In patients with type 2 DM, the expression of NLRP3 in renal tubular epithelial cells is significantly enhanced, and the levels of IL-1β and IL-18 are also significantly elevated [31]. Hyperglycemia can activate the NLRP3 inflammasome and mediate the inflammatory response of patients with DKD by ATP-P2X4 signaling, suggesting that this pathway may serve as an anti-inflammatory therapeutic target for delaying DKD [31]. Here, we found that high glucose concentrations activated the NLRP3 inflammasome in glomerular mesangial cells in a time-dependent manner, increasing the expression of IL-1β and IL-18 downstream.

MCC950, which is also known as CP-456773, is a specific blocker of NLRP3. Recently, the O’Neill group published a detailed study of MCC950 [32]. Further studies have shown that MCC950 can effectively inhibit NLRP3-related immune diseases [33]. To clarify whether high glucose concentrations increased the expression of IL-1β and IL-18 by activating caspase-1 via NLRP3 in rat glomerular mesangial cells, we used MCC950 to treat high-glucose groups. The expressions of NLRP3, caspase-1, IL-1β, and IL-18 in the intervention group were significantly lower than those in the HG group (*p* < 0.05), suggesting that the expression of IL-1β and IL-18 in glomerular mesangial cells under high glucose conditions is at least partially derived from the assembly of NLRP3 inflammatory complexes. These results indicate that high glucose exposure is involved in the development of DKD by activating the NLRP3-caspase-1-IL-1β/IL-18 signaling pathway.

There has also been considerable study of plant active ingredients in the treatment of DKD. Studies have indicated that *Tripterygium wilfordii* polyglycoside, ligustrazine, root saponin, and berberine have some effects, but the exact effects and associated mechanisms are still unclear [34–36]. Therefore, it is useful to explore the effective ingredients of botanical drugs that can affect DKD progression.

Naringin is a particularly interesting compound for several reasons, including its anti-cancer, anti-inflammatory, anti-oxidation, and other beneficial biological activities. Recently, there have been reports of the effects of naringin on DM and its potential complications. For instance, Kandhare et al. [24] studied the effects of naringin treatment through an experimental design that included a DM foot group and different doses of naringin (20, 40, and 80 mg/kg). They found that naringin treatment significantly reduced glycosylated hemoglobin and improved insulin secretion in a dose-dependent manner, demonstrating that naringin had a dose-dependent hypoglycemic effect. During treatment with naringin combined with vitamin C in STZ-induced Wistar rats, it was found that the treatment effectively improved blood glucose and glycosylated hemoglobin, increased the activity of hexose kinase in livers and kidneys, reduced glucose-6-phosphatase and fructose 1,6-diphosphate activities, reduced lipid peroxides, and increased GSH. These results suggested that the anti-hyperglycemic effects and anti-oxidative stress effects of naringin could potentially improve DKD [37]. Ali et al. [38] reported that naringin treatment of STZ-induced rats significantly reduced blood glucose, hydrogen peroxide, and thiobarbituric acid levels while increasing insulin levels and antioxidant enzyme activity, which thus resulted in an overall increase in antioxidant capacity.

Targeting the NLRP3 inflammasome has become an intensive area of research in recent years. Small molecule inhibitors have been identified including glibenclamide, chrysanthemum lactone and its derivatives, Bay 11–7082, and MCC950. Among these, MCC950 is the most widely studied [32, 33, 39–41]. Our study also found that MCC950 can effectively inhibit the expression of NLRP3. To explore the specific mechanism of naringin effects on DKD, we focused on NLRP3 and compared our results with those incorporating MCC950. Our results indicated that naringin can effectively inhibit high expression levels of NLRP3 that are caused by high glucose levels while inhibiting caspase-1. Further, it can also decrease the secretion of IL-1β/IL-18, and this inhibition is similar to that from MCC950. Previous studies have shown that naringin can significantly reduce TNF-a, IL-1β, and IL-6 in DM rats, and that it also exhibits a potent anti-inflammatory effect [24]. However, its specific mechanism is still unknown. Our results indicate that its anti-inflammatory effect may be mediated by the NLRP3 inflammasome.

Our study confirmed that different concentrations of glucose can significantly promote the proliferation of mesangial cells in a time- and concentration-dependent manner. The results of this study were similar to those reported previously regarding the role of high glucose concentrations on mesangial cells [42]. Together, these data help explain how high glucose can promote the progression of DKD by promoting cell proliferation.

In this study, different concentrations of naringin were used to assess its effect on rat glomerular mesangial cells under high glucose conditions. The results showed that when the concentration of naringin was 10 μmol/L, inhibition of mesangial cell proliferation due to high glucose occurred, and this inhibitory effect was dose-dependent. However, with increasing naringin concentrations, its drug toxicity was significant, which provides a theoretical basis for its potential dose range.

## Conclusions

Our results confirmed that naringin can regulate the NLRP3-caspase-1-IL-1β/IL-18 signaling pathway to affect the NLRP3 inflammasome, which can improve DKD by playing an anti-inflammatory role. This study provides new insights into the nephroprotective mechanism of naringin to improve DKD by anti-inflammatory responses. However, these experiments are limited to cultured mesangial cells in vitro. Morphological and functional changes in DM animal models need to be investigated in the future to assess in vivo responses.

## Additional file

Additional file 1: Key summary points. Diabetic kidney disease (DKD) is one of the most serious chronic complications of diabetes mellitus (DM), is a strong risk factor for cardiovascular diseases, and is a major cause of end stage kidney disease. The pathogenesis of DKD is complex and there are no effective measures to treat it currently.The aim of this study was to investigate the expression of the NLRP3-inflammasome under high glucose conditions, the effects of naringin during these conditions, and elucidate the role of naringin in the pathogenesis of DKD. Our results confirmed that naringin can regulate the NLRP3-Caspase-1-IL-1β / IL-18 signaling pathway by the NLRP3 inflammasome, which can improve DKD by playing an anti-inflammatory role.This study provides new insights into the nephroprotective mechanism of naringin to improve DKD by anti-inflammatory responses. (DOCX 12 kb)

## Abbreviations

ASC: Apoptosis-associated speck-like protein containing a caspase recruitment domain
CAT: Catalase from micrococcus lysodeikticus
DKD: Diabetic kidney disease
DM: Diabetes mellitus
HG: High glucose group
HM: Mannitol osmotic pressure control group
IL-18: Interleukin-18
IL-1β: Interleukin-1β
LPS: Lipopolysaccharides
MCP-1: Monocyte chemotactic protein-1
NG: Normal control group
NLRP3: Nucleotide binding and oligomerization domain-like receptorfamily pyrin domain-containing 3
SOD: Superoxide dismutase
TNF-a: Tumor necrosis factor-a
