# Supplementary material for: Naringin ameliorates the high glucose-induced rat mesangial cell inflammatory reaction by modulating the NLRP3 Inflammasome
Source: BMC Complement Altern Med. 2018 Jun 22;18:192. doi: 10.1186/s12906-018-2257-y (PMC6014005; doi:10.1186/s12906-018-2257-y)
Supplement: Supplementary file 1 — Key summary points. Diabetic kidney disease (DKD) is one of the most serious chronic complications of diabetes mellitus (DM), is a strong risk factor for cardiovascular diseases, and is a major cause of end stage kidney disease. The pathogenesis of DKD is complex and there are no effective measures to treat it currently.The aim of this study was to investigate the expression of the NLRP3-inflammasome under high glucose conditions, the effects of naringin during these conditions, and elucidate the role of naringin in the pathogenesis of DKD. Our results confirmed that naringin can regulate the NLRP3-Caspase-1-IL-1β / IL-18 signaling pathway by the NLRP3 inflammasome, which can improve DKD by playing an anti-inflammatory role.This study provides new insights into the nephroprotective mechanism of naringin to improve DKD by anti-inflammatory responses. (DOCX 12 kb) [file 12906_2018_2257_MOESM1_ESM.docx]

Diabetic kidney disease (DKD) is one of the most serious chronic complications of diabetes mellitus (DM), is a strong risk factor for cardiovascular diseases, and is a major cause of end stage kidney disease. The pathogenesis of DKD is complex and there are no effective measures to treat it currently.The aim of this study was to investigate the expression of the NLRP3-inflammasome under high glucose conditions, the effects of naringin during these conditions, and elucidate the role of naringin in the pathogenesis of DKD. Our results confirmed that naringin can regulate the NLRP3-Caspase-1-IL-1β / IL-18 signaling pathway by the NLRP3 inflammasome, which can improve DKD by playing an anti-inflammatory role.This study provides new insights into the nephroprotective mechanism of naringin to improve DKD by anti-inflammatory responses.
